# Results of Stenting for Central Venous Occlusions and Stenoses in the Hemodialysis Patients

**DOI:** 10.3400/avd.oa.20-00114

**Published:** 2020-09-25

**Authors:** Daihiko Eguchi, Kenichi Honma

**Affiliations:** 1 Department of Vascular Surgery, Fukuoka City Hospital

**Keywords:** vascular access surgery, central vein, endovascular treatment, bare metal stent

## Abstract

**Objectives**: We aim to investigate the results of stenting for central venous occlusions and stenoses in the hemodialysis patients.

**Methods:** Twenty-nine cases treated with endovascular recanalization with deployment of bare metal stent (BMS) for central venous occlusions (24 cases) and recurrent stenoses (5 cases) between 2014 and 2018 were retrospectively analyzed. Results of these procedures including success rate, operative time, estimated blood loss, morbidity, primary patency, assisted primary patency and freedom from target-lesion revascularization (TLR) were evaluated.

**Results**: Nine lesions were in brachiocephalic vein (Occlusion/Stenosis: 8/1) and 20 lesions were in subclavian vein (Occlusion/Stenosis: 16/4). Procedural success was 94% (29/31 cases) and operation time/estimated blood loss was 68±39 min/28±54 g. Symptom were relieved or disappeared in all successful cases. Morbidity (extravasation of contrast medium) was 3% (1/29). During the period of observation, 1 stent fracture with occlusion and 1 stent migration to periphery were recognized. 1-year primary patency, freedom from TLR, and assisted primary patency were 40% (median patent time: 256 days), 67% (median patent time: 524 days), and 77%, respectively.

**Conclusion:** Stenting for central venous occlusions and stenoses in the hemodialysis patients is safe and durable treatment option. However, considering its off-label use and potential hazard including vessel rupture, stent migration, and stent fracture, the indication for BMS deployment should be conservative, and interventionist should be well acquainted with prevention and measures to these complications. (This is a translation of Jpn J Vasc Surg 2019; 28: 193–198.)

## Introduction

Venous hypertension caused by central venous lesions (stenosis or occlusion) is often encountered in dialysis patients, which occur in 16%–50% of dialysis patients.^1)^ Treatment options for central venous lesions include surgical procedures such as access abandonment,^2)^ surgical revascularization (decompression of thoracic outlet), and endovascular treatment. The less invasive endovascular treatment approach is usually preferred.^3)^ In Japan, bare-metal stent (BMS) placement for central venous lesions is off-label; therefore, there are only a few reports on the results of this approach. In this study, we reported the results and patency following BMS placements for central venous lesions in symptomatic patients.

## Subjects and Methods

From November 2014 to January 2019, 62 patients with central venous lesions (stenosis or occlusion) underwent endovascular procedures; 31 received plain old balloon angioplasty (POBA) and two cases were unsuccessful. The remaining 29 cases (26 patients) underwent BMS placement and were the subjects of the study. **Table 1** shows the characteristics of these 26 patients. Brachiocephalic vein and subclavian vein were defined as central vein. Some studies include the cephalic arch as a central vein, but the cephalic arch lesions were excluded in this study. No patient had a history of subclavian venous catheter insertion at medical interviews, which is a risk factor for this disease. All dialysis patients with symptoms of venous hypertension (upper extremity edema, increased venous pressure, extended time for hemostasis, etc.) were instructed to select one of the treatment options (access abandonment, surgical repair, and endovascular treatment), and only one patient chose access abandonment followed by long-term catheter placement because of the social indications. All remaining patients chose endovascular treatment.

**Table 1:** Table 1 Patients’ characteristics

Patients characteristics	
Sex	Male 17 Female 9
Age	71±10
HD duration (days)	4326±2830 days
	median: 3049 days
Cause of HD	n (%)
CGN*	7 (27)
HTN**	6 (23)
DMN***	5 (19)
Others	8 (31)
Comorbidity	
Hypertension	18 (69)
Cerebrovascular disease	7 (27)
Diabetes mellitus	6 (23)
Ischemic heart disease	5 (19)
Chronic heart failure	3 (12)
Lesion (occlusion/stenosis)	
Brachiocephalic	8/1
Subclavian	16/4

*CGN: chronic glomerulonephritis; **HTN: hypertensive nephropathy; ***DMN: diabetic nephropathy

**Figure 1:**
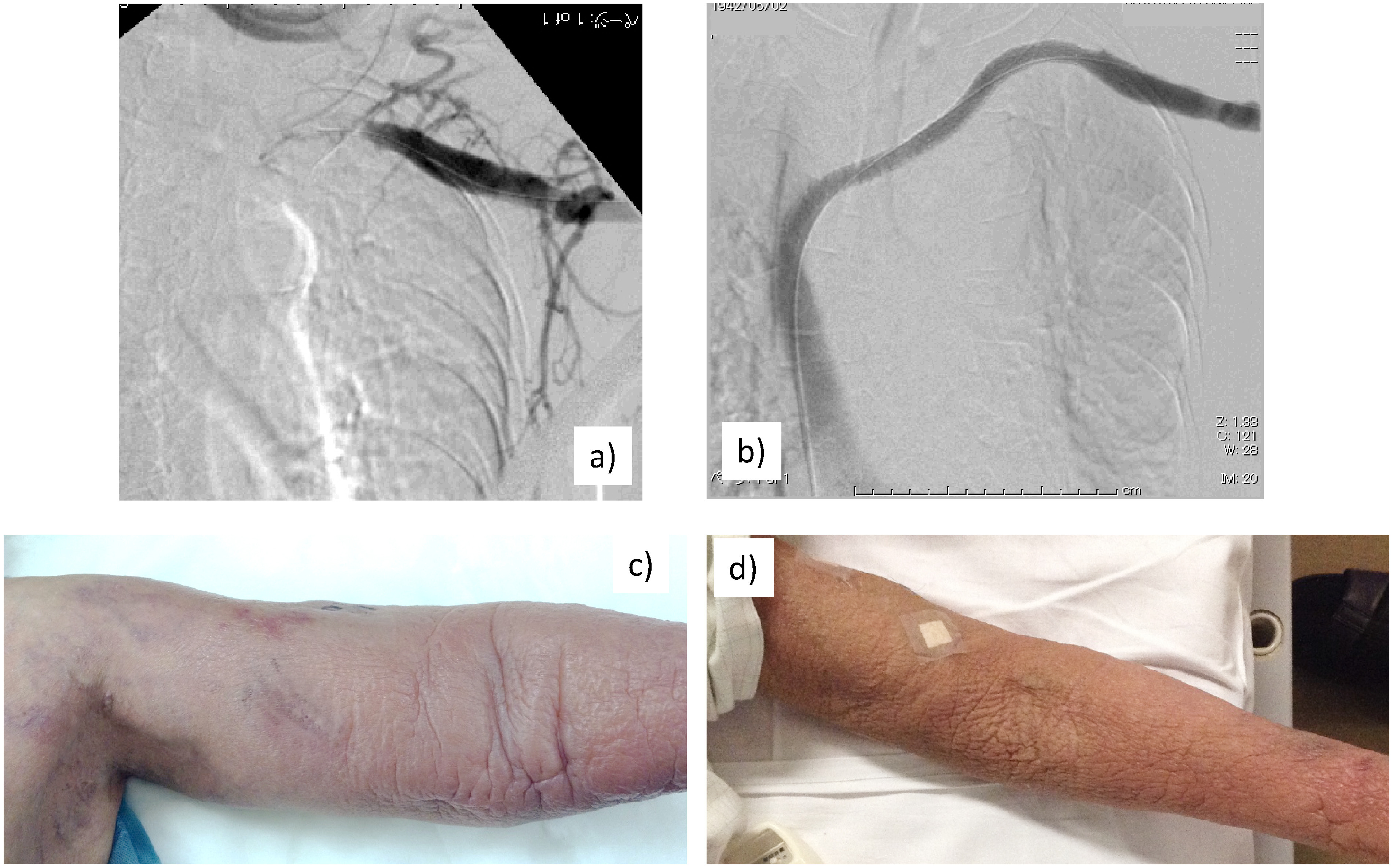
Fig. 1 (**a**) Preoperative angiography shows occluded left subclavian and brachiocephalic vein. (**b**) Postoperative angiography shows recanalization of occluded vein with BMS. (**c**) Preoperative patient’s left arm. Note disabling edema of patient’s left arm. (**d**) Postoperatively, patient’s edematous left arm was reduced to normal.

The endovascular treatment procedures were as follows: A 6-F sheath was inserted through the access circuit under local anesthesia and the angiography was performed. The central venous lesion was confirmed and the guide wire (GW) approached. For lesions in the subclavian vein, the GW was advanced through the basilic vein because the torque sometimes does not get transmitted sufficiently due to the angle of the cephalic arch confluence, if the cephalic vein approach is used. As a back-up measure, it was also useful to insert sheaths from both the cephalic and basilic sides and to get angiography from both sides. The GWs used were 0.018 or 0.035 inches in diameter and crossed the lesions. Predilatation was performed using 4- or 5-mm balloons. The BMSs were then placed and postdilation was performed using 8–12-mm balloons. Size of BMSs were 1 or 2 mm larger than reference vessel diameter, and the size of postdilatation balloon was approximately the same as reference vessel diameter. Postdilatation balloon pressure was maintained as low as possible relative to the extent of expansion. For long brachiocephalic vein occlusions in which the device could not pass using a monodirectional approach, a bidirectional approach from upper limb and right femoral vein was used and a tug of wire was established to facilitate device passage. **Figure 1** shows the pre- and postoperative images of a case of BMS placement for occluded brachiocephalic vein.

No postoperative anticoagulant therapy was administered nor was regular surveillance performed. Patients with findings or symptoms of increased venous pressure or upper limb edema were referred by a maintenance dialysis facility and underwent angiography or percutaneous transluminal angioplasty (PTA) or other procedures, if necessary.

All data are expressed as mean±standard deviation. Based on the definition of patency of vascular access by Gray et al.,^4)^ primary patency was defined as interval following intervention until the next reintervention (including both the target lesion and all access circuit starting from the arterial anastomosis to the superior vena cava). Primary-assisted patency was defined as interval after intervention until access thrombosis. The rate of freedom from TLR was defined as interval after intervention until the next reintervention at or adjacent to original central venous site. Kaplan–Meier survival curves were used for analysis.

This BMS study was not covered by national insurance and was reviewed and approved by the ethics committee (approval number: 138) of our facility. Patients were informed about the insurance non-coverage and that there was no economic disadvantage to the patient, after which consent was obtained.

## Results

**Figure 2:**
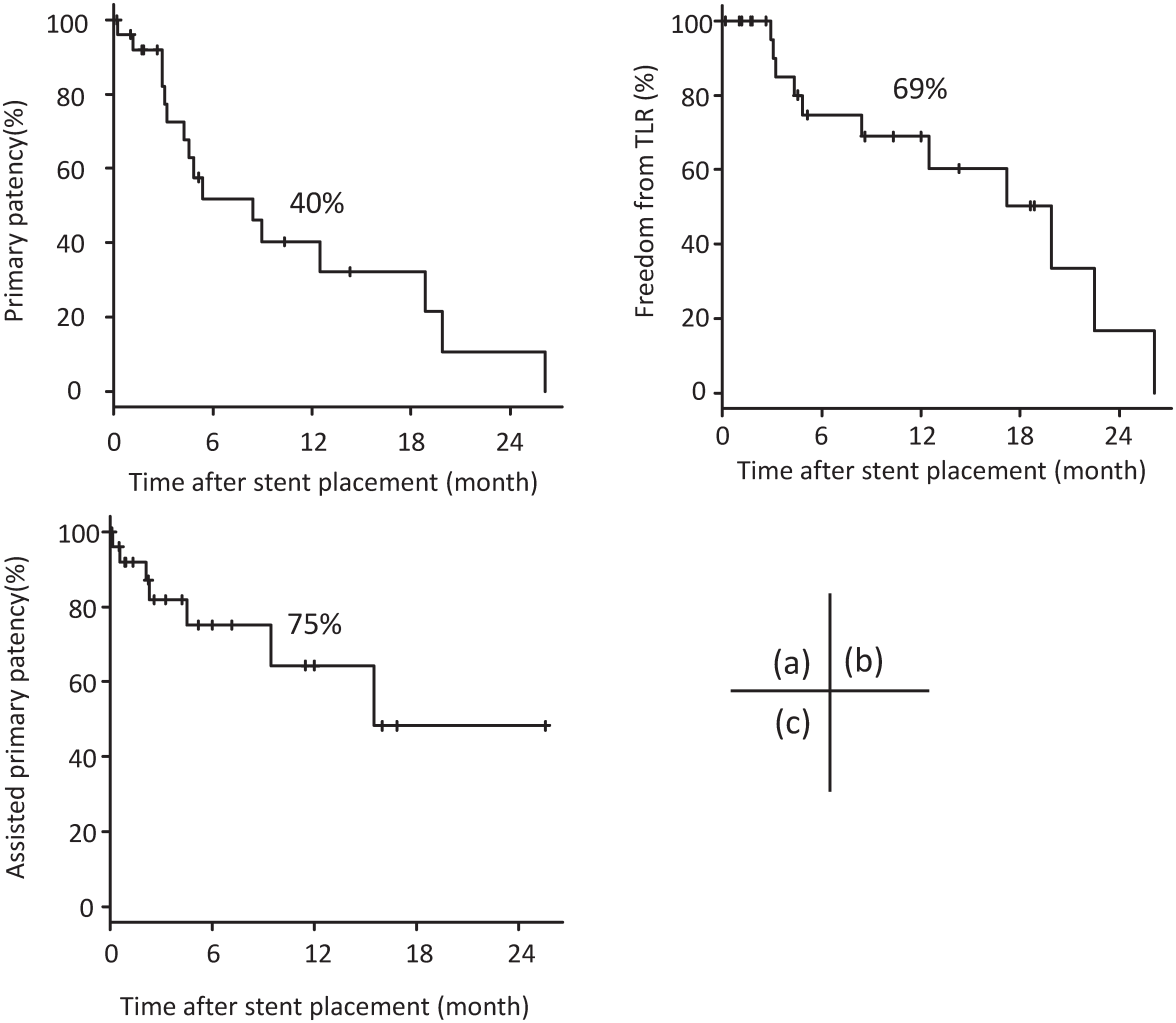
Fig. 2 (**a**) Primary patency, (**b**) freedom from TLR, (**c**) assisted primary patency, after BMS placement in central vein. Figures in each graph are results of 1 year.

The results are shown in **Table 2**. Procedure time was 68±38 min, with estimated blood loss was 28±54 g, and 97% of the BMS placed were self-expanding stents. A balloon-expanded stent was used to expand the stent strut in a case of stenosis caused by the migration of a self-expanding stent. Of the 29 cases, three were case of secondary BMS placement: one case of occlusion due to stent fracture, one case of occlusion due to in-stent intimal hyperplasia, and one case of stenosis due to stent migration. Complications during the procedure occurred in 1/29 cases (3%). In this case, extravasation of contrast media occurred, but hemostasis was achieved by additional BMS placement and balloon tamponade. The procedural success rate was 94% (29/31), and symptoms were relieved or disappeared in all successful cases. Long-term complications included a stent fracture (occlusion) and a stent migration (from the subclavian vein to the axillary vein). Primary patency after 1 year was 40% (median patent period, 256 days), and assisted primary patency was 75%. Rate of freedom from TLR after a year was 69% (median patent period, 606 days; **Fig. 2**).

**Table 2:** Table 2 Operative results (29 cases)

Time (min)	68±39 min
Blood loss (g)	28±54 g
BMS	
Self-expandable stent	
7–9 mm	8 (28)
10 mm	12 (41)
12–14 mm	8 (28)
Balloon-expandable stent	
8 mm	1 (3)
Intraoperative complication	
Extravasation of contrast medium	1/29 (3)
Initial success (%)	29/31 (94)
Late complication	
Stent fracture (occlusion)	1/29 (3)
Migration (stenosis)	1/29 (3)

n (%)

## Discussion

Central venous lesion (stenosis/occlusion) are frequent complications in dialysis patients, and many patients complain of unpleasant symptoms associated with venous hypertension (upper limb edema, dilation of superficial veins, etc.).^2)^ While the causes of central venous lesions are unclear, changes in shear stress due to increased blood flow, increased blood flow velocity, turbulence, and oxidative stress may lead to endothelial damage.^2)^ In addition, intimal thickening may occur due to inflammation and venous injury caused by prior catheter placement. Although venous thoracic outlet syndrome^5)^ may be involved, the incidence of this syndrome is extremely rare (1–2 persons in 100,000 population) and is more common in young men in their 30s.^6)^ Thus, thoracic outlet syndrome is unlikely to contribute to pathogenesis of central venous lesions in dialysis patients. Compression of the subclavian vein between the clavicle and the first rib is a phenomenon that is observed even in healthy subjects when the upper limb is abducted.^6)^ Hence, this physiological subclavian vein compression are not considered to cause venous hypertension. However, when BMS placement is performed on central venous lesions, attention should be paid to these anatomical factors because they may contribute to complications such as stent deformation and occlusion (described later). Catheter placement through the subclavian vein is a major risk factor for central venous lesions^7)^; however, according to the medical interviews, there were no catheter placements through the subclavian vein in the patients’ history in this study. This fact indicates a high level of recognition of this lesion among nephrologists.

Treatment for symptomatic central venous lesions is roughly divided into access abandon, open surgical repair, and endovascular treatment. In our department, our policy is to perform endovascular treatment unless patients and/or their family chose access abandon and tunneled dialysis catheter insertion because of disabled activities of daily living and difficulty in visiting our hospital. Therapeutic intervention for asymptomatic central venous lesions is reported to be futile,^8)^ and we do not perform interventions for asymptomatic central venous lesions.

Although we perform primary BMS placement for symptomatic central venous occlusion, for stenotic lesions, we usually perform POBA. Only for frequent recurrent stenotic lesions, we consider BMS placement. As for surgical repair, bypass to internal jugular vein or brachiocephalic vein, and/or decompression of the thoracic outlet have been reported, but considering the high degree of surgical invasion and recurrence rates, it is hard to say that these are ideal treatments.^7,9)^ Although the results of POBA for central venous stenosis are reported to be equivalent to those of BMS placement,^10)^ which cannot apply to cases of central venous occlusion. Considering the safety at the time of recanalization and the concern about high recoil rates after POBA,^11,12)^ we believe that primary BMS placement is more effective and safer in cases of central venous occlusion. Stent grafts which became available in the peripheral arterial lesions in Japan are known to perform better than BMS used in central venous lesions in terms of patency,^13)^ however, we refrain from using these because of concerns regarding occlusion of the internal jugular vein or the cephalic vein at their confluence and the need for large-diameter sheaths (11-F sheath is required for 10 mm stent grafts). Although we believe that stent grafts are effective in occluded or stenosed cephalic arch where there is no important branch with a thin diameter, there are few advantages for stent grafts in central venous lesions.

Vascular injury (cardiac tamponade)^14)^ and migration of stents into the right atrium or pulmonary artery^15)^ are serious complications during or immediately after BMS placement. In our case, a small amount of extravasation after recanalization and balloon dilatation of an occlusion was observed in a patient following stent fracture. Balloon tamponade for hemostasis was required, but when vascular injury is serious and massive bleeding occur, ligation of inflow vessel or stent graft deployment (in principle off-label) should also be considered. In addition, migration of the stent into the right atrium or pulmonary artery trunk is a serious complication that sometimes requires open heart surgery.^16)^ Selection of a stent with sufficient diameter to prevent the stent from falling downstream and placement of the stent at a sufficient length upstream of the lesion to anchor it are necessary.^12)^

Regarding the type of stent, balloon-expanded stents cannot cope with changes in venous diameter due to changes in venous flow. Furthermore, the risk of occlusion and migration is high because compression from outside the blood vessels produces irreversible deformation.^17,18)^ Therefore, the use of self-expanding stents is common.^19)^ In case of migration into the superior vena cava or right atrium, a balloon or snare from the femoral vein (bidirectional approach) may be used to retrieve the migrated stent, bridge it to the inferior vena cava and the superior vena cava, or place it in the internal iliac vein.^15)^ In any case, if the GW is secured in the stent lumen, the above-mentioned treatment can be immediately performed; therefore, a sufficient length of GW ahead of the lesion should be inserted.

We evaluated the long-term results of the study. The primary patency, freedom from TLR, and assisted primary patency after 1 year was 40%, 69%, and 75%, respectively. The difference between the primary patency and the freedom from TLR depends on the presence or absence of up-stream lesions accompanied by the central venous lesions. In other words, the central venous lesions only worsen symptoms of venous hypertension without hindering normal dialysis performance. The reported patency after BMS placement for central venous lesions are variable. Kundu et al. reported a 1-year primary patency of 14%–73%^20)^ for central venous lesions. In 2016, Kang et al.^21)^ reported 401 cases of central venous lesions (164 cases of occlusion and 237 cases of stenosis), the highest number of cases reported to date. The 1-year primary patency in this study ranged from 16%–28.8%, and the median patency period was 8.5–10.9 months. Actually, the “primary patency” referred by them correspond to the “freedom from TLR” in our study. Therefore, it may well be considered that we achieved better results than previous studies. As Gray et al. pointed out that confusion in definitions of patency is relatively common in the literature of vascular access interventions, and this should be noted when comparing and interpreting their results.^4)^ In addition to the confusion associated with the definition of patency, the nature of the central venous lesion itself could influence the variation in patency. As mentioned above, central venous lesions often progress silently without affecting dialysis performance even when they recur, so we cannot deny that in facilities like ours that perform therapeutic interventions only when symptoms recur or when dialysis performance is affected, primary patency and freedom from TLR tend to be better than in facilities that regularly do surveillance by angiography or ultrasound. Long-term complications include stent migration, stent fracture, and stent infection, but to the best of our knowledge, there are no reports on central vein stent infection. A case of stent fracture was observed in our study. To date, precise frequencies of BMS fractures after placement in central veins have not been reported. Physiologically, the brachiocephalic vein is almost closed in its course between the sternum and aortic arch during normal inspiration,^22)^ and the subclavian vein is markedly narrowed at the thorax outlet during upper limb abduction. Considering these facts, the possibility of migration or fracture should always be considered during BMS placement at this area. Usually, a secondary BMS placement is required for stent fracture cases. In a study reporting 29 cases of secondary BMS placement, the 1-year primary patency (32%) after a secondary stent placement was not different from that after the first stent placement (26%). Thus, acceptable patency can be expected to some extent.^1)^ However, when the secondary stent also encounters problems, such as fractures, bypass surgery or access abandonment may eventually be necessary. Further studies on long-term results as well as the development of a new BMS that is flexible enough to resist kinking and fracture are needed.

## Conclusion

Although the treatment results and patency of BMS placement for central venous lesions in dialysis patients with symptoms of venous hypertension are considered good and effective, clinicians who perform this intervention must recognize that BMS placement in vascular access intervention is off-label, and there is the remote possibility of vascular injury, stent migration, and stent fracture.

## Notes

This is a translation of Jpn J Vasc Surg 2019; 28: 193–198.
